# Combined effects of vitamin D supplementation and endurance exercise training on insulin resistance in newly diagnosed type 2 diabetes mellitus patients with vitamin D deficiency: study protocol for a randomized controlled trial

**DOI:** 10.1186/s13063-021-05861-x

**Published:** 2021-12-06

**Authors:** Mi Xiang, Xiaomin Sun, Junxiang Wei, Zhen-Bo Cao

**Affiliations:** 1 School of Public Health, Xinhua Hospital, Shanghai Jiao Tong University, Shanghai, 200025 China; 2 Global Health Institute, School of Public Health, Xi’an Jiaotong University Health Science Center, 76 Yanta West Road, Xi’an, Shaanxi, 710061 China; 3 Shanghai Frontiers Science Research Base of Exercise and Metabolic Health, School of Kinesiology, Shanghai University of Sport, 399 Chang Hai Road, Shanghai, 200438 China

**Keywords:** Type 2 diabetes mellitus, Insulin resistance, Vitamin D, Endurance exercise

## Abstract

**Background:**

Although approximately 50% of Chinese with type 2 diabetes mellitus (T2DM) patients have vitamin D deficiency, studies regarding vitamin D supplementation on insulin resistance (IR) have mainly focused on non-Asians. Endurance exercise training (ET) enhances insulin-mediated glucose metabolism, which plays a critical role in T2DM prevention and control. However, the combined effects of vitamin D supplementation and ET on IR in T2DM patients are unclear. The objectives of this study is to investigate the synergistic effect of vitamin D supplementation combined with exercise training intervention on IR in T2DM patients.

**Methods and analysis:**

We propose a 3-month randomized controlled trial among 60 T2DM patients aged 40–65, newly diagnosed with T2DM ≤ 1 year, and with stable HbA1c level (≤ 8.0%) in the past 3 months. The participants will be randomly allocated to the vitamin D group, vitamin D combined with exercise training group, exercise training group, and control group (CG) using a computer-generated random number sequence. At baseline, participants will undergo a medical review, anthropometric measurements, dual X-ray absorptiometry, a 75-g oral glucose tolerance test (OGTT), ankle-brachial index measurements, and physical fitness measurements and will complete related lifestyle questionnaires. Fasting blood lipid and glucose levels were also measured. In a 3-month intervention period, vitamin D intervention group will receive a dose of 1000 IU daily; exercise group will perform a 1-h endurance exercise 3 times per week (maximal heart rate, 60–80%), and the control group will receive apparently identical tablets. Additionally, all participants will be advised to maintain their normal diet and physical activities during the intervention. All measurements will be repeated at 3-month follow-up after the intervention with the primary outcome measure expressed as a change from baseline in insulin sensitivity and secretion. Secondary outcome measures will compare the changes in anthropometry, ankle-brachial index, and physical fitness factors (e.g., peak oxygen uptake, hand grip strength). Data will be managed and analyzed using the Statistical Package for the Social Sciences.

**Discussion:**

This is the first study to conduct a randomized trial to clearly determine the independent and combined effects of vitamin D supplementation and endurance exercise trial on IR in Chinese T2DM patients as measured by OGTT. The findings from the proposed study will not only provide new evidences that vitamin D supplementation plays an important role in IR management but also develop a simple and efficient method to improve IR-associated metabolic diseases for T2DM patients.

**Trial registration:**

Chinese Clinical Trial Registry ChiCTR1800015383, Registered on 28 March 2018

## Introduction

With rapid social and economic developments during the past three decades, China is facing a growing threat from non-communicable chronic diseases (NCDs), with diabetes being considered one of the most common NCDs in China and several countries [1]. Diabetes prevalence in Chinese adults increased substantially from 0.67% in 1980 to 11.6% in 2010 and 10.9% in 2013 [2]. Insulin resistance (IR) is considered a prominent feature of type 2 diabetes mellitus (T2DM) and an important pathological and physiological basis for the development of T2DM [3]. How to effectively improve IR has become one of the emphases in the treatment of T2DM.

Vitamin D deficiency [25(OH)D < 20 ng/mL] is prevalent in several populations and has become a common public health problem worldwide due to sun protection measures, reduction of outdoor activities, increased obesity prevalence, and environmental pollution [4]. Zhang and colleagues recently reported that in China, 50% of T2DM patients were vitamin D deficient, further deteriorating their glucose tolerance status [5].

Mounting evidence suggests that altered vitamin D homeostasis may play a role in the development of IR and T2DM [6]. A prospective cohort study and meta-analysis indicated that subjects with 25-hydroxyvitamin D [25(OH)D] levels < 20 ng/mL were more likely to be diagnosed with T2DM compared to subjects with 25(OH)D levels ≥ 20 ng/ml [7]. However, to date, studies on vitamin D supplementation on IR in T2DM patients have mainly focused on non-Asian countries, and the results are inconsistent [8–13].

Krul-Poel et al. conducted a 6-month randomized controlled trial of vitamin D supplementation (50,000 IU/month) in 275 T2DM patients and found that glycated hemoglobin (HbA1c) levels did not improve in individuals with vitamin D < 20 ng/mL [8]. Similar results were also observed in 86 German T2DM patients with dose of 1904 IU/d for 6 months [9], while von Hurst et al. [14] found that 6 months of daily vitamin D supplementation could significantly reduce HOMA-IR values in vitamin D-deficient South Asian women living in New Zealand. The results were also supported by our previous study in Japanese adults [15]. The disparity may be partly attributed to the ethnicity-related differences in vitamin D receptor (VDR) polymorphisms (e.g., Apa I and Fok I) and insulin sensitivity [16, 17].

Documented evidence demonstrated that physical inactivity and obesity are two major risk factor for the development of T2DM; exercise intervention, where aerobic or resistance training or a combination, can exert many beneficial effects, such as increase fat oxidations, in the prevention and treatment of T2DM [18]. Exercise training not only increases energy consumption and reduces the accumulation of lipids in insulin-sensitive tissue such as the skeletal muscle [19] but also promotes glucose transportor-4 (GLUT-4) translocation and glucose uptake in skeletal muscle cells by activating AMP-activated protein kinase (AMPK) and downstream proteins of insulin-transmitting signals in skeletal muscle cells [20]. Additionally, recent studies have shown that exercise may also be involved in the regulation of vitamin D, which can increase VDR expression in skeletal muscle tissue and serum 25(OH)D levels [21–23]. Prior evidence suggested that in addition to regulating Ca^2+^ levels, vitamin D supplementation can directly upregulate the AMPK-GLUT-4 signaling pathway through VDR related (phosphorylated ERK1/2 and Mnk1) expression to increase glucose utilization and participate in exercise pathways of glucose utilization pathways [23]. These results suggested that vitamin D supplementation combined with exercise intervention may have a synergistic effect on the improvement of IR by activating different glucose utilization pathways. However, studies investigating the synergistic effect of vitamin D supplementation combined with exercise training intervention on IR in T2DM patients are relatively few [24].

In summary, this study aimed to (1) examine the effect of vitamin D supplementation on IR and (2) determine the synergistic effect of vitamin D supplementation combined with exercise training intervention on IR in Chinese T2DM patients with vitamin D deficiency by a randomized controlled trial.

## Methods and analysis

### Study design

**Fig. 1:**
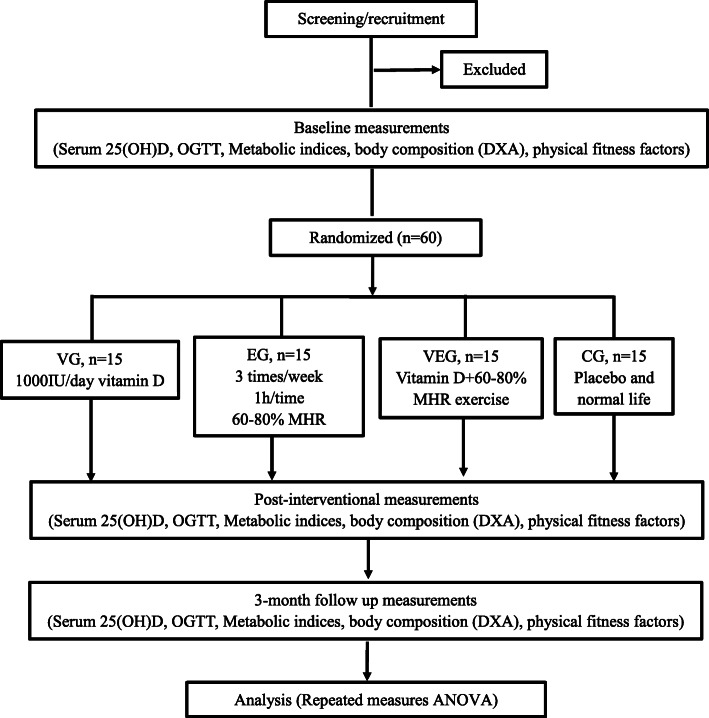
Study design flow chart. OGTT, oral glucose tolerance test; DXA, dual X-ray absorptiometry; VD, vitamin D; EG, endurance exercise group; VEG, vitamin D and endurance exercise group; CG, control group; MHR, maximal heart rate; ANOVA, analysis of variance

The study is a randomized controlled trial with a 3-month intervention and 3-month follow-up period assessing the impact of combined vitamin D and exercise intervention on improving glucose and lipid metabolism in T2DM patients in Xi’an, China (34° N latitude). Vitamin D or placebo supplementation is designed as a double-blind trial. Sixty T2DM patients aged 40–65 years with serum 25(OH)D concentrations < 20 ng/mL on screening will be enrolled during winter and spring through posters and WeChat. Patients will be randomly assigned to either an intervention or a control group. The complete process is outlined in Fig. 1.

### Setting and participants

#### Inclusion and exclusion criteria

T2DM patients are eligible for study participation if they meet the following inclusion criteria: (a) patients newly diagnosed with T2DM ≤ 1 year, (b) patients with HbA1c level remaining stable at ≤ 8.0% in the past 3 months and patients with no plan of replacing the hypoglycemic agent in the near future, (c) patients without regular vitamin D and/or calcium supplements in the past year, (d) patients who do not meet the current National Physical Activity guidelines [25] in the past year, and (e) with stable body weight (≤ 2 kg variation in weight within the last 3 months.

The following participants were excluded: participants with acute infection; participants experiencing stress; participants experiencing the acute complications of diabetes; participants with heart, liver, and kidney insufficiency, osteoporosis and fracture, and metal implants in the body that could affect magnetic resonance imaging and dual-energy X-ray absorptiometry (DXA) measurements; and participants who have used insulin therapy and have history of sunbathing in the last 6 months.

The criteria for early termination of intervention are as follows: (a) blood calcium level is abnormally high (> 2.65 nmol/L), (b) serum 25(OH)D levels are abnormally high (> 100 ng/mL), and (c) have plan to replace with stronger blood glucose lowering drugs or HbA1c levels are > 8.5%.

### Randomization

All patients who give consent for participation and who fulfill the inclusion criteria will be randomized. An investigator who is independent of this study will randomly divide the subjects into four groups according to a computer-generated random allocation table stratified by age and sex. The treatment allocation code will be placed in an opaque envelope labeled with a sequential number. Group allocation was unknown to participants, investigators, and data collectors. The randomization code was broken after the last participant finished the study. The four groups are as follows: the placebo control group (CG), vitamin D supplementation group (VG), endurance exercise group (EG), and vitamin D supplementation combined endurance exercise group (VEG). All patients will be instructed not to change their levels of general physical activity and dietary habits during the intervention period. Their dietary changes will be recorded monthly as well.

### Intervention procedure

#### Vitamin D intervention

Patients in the VG or VEG group will receive one tablet of vitamin D_3_ supplement (1000 IU/day, Nature Made, Otsuka Pharmaceutical Co, Ltd, Tokyo, Japan) immediately after meals to enhance absorption based on the guideline of Institute of Medicine for Vitamin D recommended [26], and patients in the EG or CG group will receive an identical appearance shape and color placebo as the vitamin D_3_ supplement every day for 3 months.

#### Exercise intervention

Patients in the EG or VEG group will perform a 1-h progressively increasing aerobic exercise (cycling, running, or rowing) at 60–80% of maximal heart rate 3 times a week for 3 months [25], and this will be supervised by a qualified trainer who is knowledgeable of the study protocol and procedures. Polar monitor will be used to monitor heart rate during exercise, and the compliance (exercise time and intensity) with each protocol will be recorded. Participants will warm up during the first 5 min on a treadmill at 50–60% of maximal heart rate and subsequently follow the exercise protocol assigned to them, with a 5–10-min recovery exercise at 40–50% of maximal heart rate, which comprises walking and stretching exercise.

### Patient and public involvement

Patients will not be involved in recruitment of participants or conduct of the study. The results of this study will feedback and disseminate to all of participants.

### Data collection

#### Outcome measures and measurement procedures

A full list of measurable outcomes is presented in Table 1. Participants will be assessed at baseline, at the end of intervention, and 3 months after the intervention (Table 2).

**Table 1:** Summary of outcome measures

Outcomes	Item	Device
**Primary outcomes**		
Insulin sensitivity and insulin secretion	The Matsuda index, the insulinogenic index, HOMA-IR, HOMA-β	OGTT
		Fasting blood measurements
Vitamin D level	Serum 25(OH)D, 1,25(OH)_2_D	Fasting blood measurements
**Secondary outcomes**		
Body composition	Percent body fat, muscle mass	DXA
	Waist circumference	Standard tape
Metabolic indices	Fasting glucose (G_0_), fasting insulin (I_0_), HbA1c, triglyceride, cholesterol, low-density lipoprotein, and high-density lipoprotein	Fasting blood measurements
Ankle-brachial index	Blood pressure, ABI, baPWV	Validated automatic device
Physical fitness factors	Peak oxygen uptake, maximal heart rate	Cycle ergometer
	Hand grip strength	Hand grip dynamometer

*HOMA* Homeostatic model assessment, *IR* Insulin resistance, *25(OH)D* 25-hydroxyvitamin D, *1,25(OH)*_*2*_*D* 1,25-Dihydroxyvitamin D, *OGTT* Oral glucose tolerance test, *DXA* Dual-energy X-ray absorptiometry, *HbA1c* Glycated hemoglobin, *ABI* Ankle-brachial index

**Table 2:** Example template of recommended content for the schedule of enrolment, interventions, and assessments

	Study period				
Timepoint	Enrolment and baseline allocation 0 week	Intervention 1–12 weeks	Post-intervention 13 weeks	Follow-up 13–24 weeks	Close-out 25 weeks
**Enrolment**					
Eligibility screen	X				
Informed consent	X				
Randomization and allocation	X				
**Exercise/vitamin D intervention**		X			
**Assessment**					
Blood parameter tests	X		X	X	
Body composition	X		X	X	
Ankle-brachial index	X		X	X	
Physical fitness factors	X		X	X	

Analysis of blood samples is as follows: a standard 75-g oral glucose tolerance test (OGTT) will be performed between 0830 and 1100 after a 12-h overnight fast, and venous blood samples will be collected in Venoject-II AutoSep tubes at time points 0, 30, 60, 90, and 120 min to determine the plasma glucose and serum insulin levels. Fasting serum sample will be used to measure the levels of 25(OH)D, 1,25(OH)_2_D, calcium, fasting glucose (*G*_0_), fasting insulin (*I*_0_), HbA1c, triglyceride, cholesterol, low-density lipoprotein, and high-density lipoprotein.

Primary outcomes are as follows: changes in insulin sensitivity and secretion.

Fasting blood glucose and insulin will be used to calculate the insulin resistance index (Homeostatic Model Assessment of Insulin Resistance [HOMA-IR]) and β cell secretion index as follows: HOMA-IR = *G*_0_ × *I*_0_/22.5 and HOMA-β = 20 × *I*_0_/(*G*_0_ – 3.5).

Insulin sensitivity estimated using the Matsuda index of insulin sensitivity during the OGTT will be calculated as follows: 1000/square root of ([*G*_0_ × *I*_0_]) × (mean OGTT glucose concentration × mean OGTT insulin concentration) [27]. Early phase of insulin secretion will be estimated using the insulinogenic index as follows: *I*_30_–*I*_0_/*G*_30_ – *G*_0_ (△I30/△G30), where *I*_30_ and *G*_30_ represent insulin and glucose values at 30 min during the OGTT, respectively [28]. The increments in the area under the curves during the complete 120-min period of the OGTT will be calculated using the trapezoid rule to assess the total changes in glucose and insulin levels [22].

Secondary outcomes are as follows: changes in anthropometry, metabolic indices, ankle-brachial index, and physical fitness factors

#### Anthropometry

Height and body mass will be measured with the participants in light clothing and barefoot. Body mass index will be calculated by dividing the body mass in kilograms by the square of height in meters (kg/m^2^). Waist circumference will be measured to the nearest 0.1 cm at the umbilical region using an inelastic measuring tape at the end of normal expiration. DXA is used to measure percent body fat and muscle mass (Hologic QDR-4500, DXA Scanner, Hologic Inc., Waltham, MA, USA) by a recognized technologist.

#### Ankle-brachial index

Blood pressure and brachial-ankle pulse wave velocity (baPWV) will be measured after the participant had rested supine for at least 5 min using a validated automatic device (BP-203RPE, II form PWV/ABI, Omron-Colin, Japan) in the brachial and ankle arteries. An oscillometric method will be used, and its cuffs have sensors that transmit data to the device. The baPWV will be calculated by dividing the distance from the aortic valve to the ankle artery by the sum of the time between the aortic valve closing sound and the notch of the brachial pulse wave and the time between the increase in the brachial pulse wave and that of the ankle pulse wave. The ankle-brachial index will be calculated by dividing the highest value obtained at each ankle by the highest of the arm values [29].

#### Physical fitness factors

Peak oxygen uptake (VO_2peak_) will be measured using a maximal graded exercise test using a cycle ergometer (MetaMax 3B, Cortex, Germany). The graded cycle exercise will begin at a workload of 45–90 W, which is subsequently increased by 15 W/min until the subject could not maintain the required pedaling frequency of 60 rpm. During the progressive exercise test, the expired gas of subjects will be collected, and the rates of oxygen consumption and carbon dioxide production will be measured and averaged over 30-s intervals using an automated gas analyzing system. The highest recorded value of VO_2_ and heart rate during the exercise test will be quantified as the VO_2peak_ (mL·kg^−1^·min^−1^) and maximal heart rate (bpm) [30]. Vigorous activities and alcohol and caffeinated beverages will be prohibited 2 days before the test.

Handgrip strength will be measured using a hand grip dynamometer (HK6800-WL, Shenzhen, China) in units of kilograms. Participants will be instructed to complete two handgrip contraction trials bilaterally, alternating hands between trials. The highest values obtained using each hand were considered the right-hand and left-hand grip strength scores [30]. The mean value of the two maximal grip strength scores will be used.

#### Sunlight exposure

Participants will be instructed to record their outdoor activity time and exposed areas of the skin from 9 am to 5 pm for 7 consecutive days in a week using a questionnaire every month. A score to estimate the mean weekly sunlight exposure will be calculated, and additional details were published elsewhere [31].

#### International Physical Activity Questionnaire

Daily physical activity except for the exercise program will be assessed using the International Physical Activity Questionnaire monthly and expressed as metabolic equivalent minutes per week. It refers to the preceding 7 days and instructs participants to report the number of days, hours, and minutes spent on vigorous activity, such as aerobics, or moderate activity, such as carrying light loads [32].

### Follow-up visits

Participants will be scheduled for their follow-up visits during the last week of their treatment assessing all procedures performed during baseline assessment including blood pressure measurement, anthropometry, OGTT, DXA, physical activity and dietary assessment using structured questionnaires.

### Safety and adherence considerations

Participant burden of the intervention and measures was assessed by interviews and feedback from patients who participated in previous pilot trail. Complaints and adverse events will be recorded during the intervention period. If patients have serious complications during the intervention, the participants will be asked to discontinue the trial, and the doctor will administer appropriate treatment.

The intervention content of this study was based on the guideline of the American College of Sports Medicine and Institute of Medicine, and measures will be conducted in hospital by specialist doctors and trainers. The dose of vitamin D used in the present study is expected to significantly increase serum 25(OH)D concentrations and decrease glucose levels in T2DM patients [33] and is lower than the tolerable upper dose (2000 IU/day) for Chinese [34]. During the screening, baseline, intervention, and follow-up, any medical conditions or abnormalities detected will be promptly discussed with the participant by a qualified medical practitioner involved in the study. Participants are allowed to be treated, referred, or advised to visit their practitioner during the intervention. All participants will be informed of their screening blood test results after they have completed their participation in the study.

The double data entry is performed in the study. There is no special Data Monitoring Committee, as the Xi’an Jiaotong University Human Research Ethics Committee, which is independent of the trial sponsor and investigator to ensure recommendations are as objective as possible and unaffected by conflicts of interest, undertake this analysis per year. The data used in this study are not publicly available due to ethical reasons; the corresponding author can provide further information on these data upon reasonable request.

The researcher committee will evaluate and improve the protocol through email or online regular meeting. The research ethics committee of the university will be also notified whenever needed.

### Statistical analyses

Based on the relative studies on vitamin D intervention with IR [15], the sample size required for each group was estimated to be 12 with a power of 85%, and an effect size of 0.26 was assumed. Therefore, 48 T2DM patients are required. To account for 20% loss-to-follow-up, a total of 60 T2DM patients will be recruited. Power calculations will be performed using G*Power software version 3.1.9.2 [35].

Descriptive statistics will be calculated using mean (standard deviation) for continuous variables and *n* (%) for categorical variables. Differences will be compared using one-way analysis of variance (ANOVA) for continuous variables and chi-squared tests for categorical variables. Repeated measures ANOVA will be used to test the effect of vitamin D and exercise combined intervention on glucose and lipid metabolism, with adjustment for baseline levels of outcomes. A post hoc test with Bonferroni correction will be used to identify significant differences among the mean values when a significant main effect or interaction is identified. Finally, in the event of possible losses or dropouts, a statistical analysis will be performed by protocol and intention to treat. Data will be entered and cleaned using EpiData version 3.0 and managed and analyzed using the Statistical Package for the Social Sciences. Statistical significance will be set at *P* < 0.05.

## Discussion

To the best of our knowledge, this is the first study to conduct a randomized trial to clearly determine the independent and combined effects of vitamin D supplementation and endurance exercise trial on IR in Chinese T2DM patients as measured by OGTT. The findings from the proposed study will not only provide new evidences that vitamin D supplementation plays an important role in reducing IR but also develops a simple and efficient method to improve IR and associated metabolic diseases for T2DM patients.

The number of people with T2DM worldwide has increased rapidly. It is estimated to reach over 700 million in 2045 including more than 120 million people with T2DM from China [36], which can lead to major social, health, and economic challenges. IR is not only considered an important pathological and physiological basis for the development of T2DM but also a major contributor to other complications, which would lead to atherosclerosis, myocardial infarction, stroke, and even death [37]. Hence, the effective and innovative methods of preventing and improving IR for T2DM population are urgently required.

Beyond its traditional role in maintaining bone health, higher serum vitamin D levels have also been found to be associated with low risk of developing T2DM and other metabolic diseases considering its potential effects on IR [3, 38, 39]. However, to date, the results of several studies conducted are inconsistent [8–13]. Moreover, considering the ethnic differences in insulin metabolism [40] and that studies on vitamin D supplementation on IR in T2DM patients have mainly focused on non-Asian populations, it is significantly important to investigate the effects of vitamin D on IR in Asians.

In addition to promoting GLUT-4 translocation and glucose uptake in skeletal muscle cells [21, 22], exercise training may also be involved in the regulation of vitamin D through increasing VDR expression in skeletal muscle tissue and serum 25(OH)D levels [23, 24, 41]. Meanwhile, vitamin D supplementation can directly upregulate AMPK-GLUT-4 signaling pathway through VDR to increase glucose utilization and participate in exercise pathway of glucose utilization [42]. Therefore, the combined intervention of vitamin D and exercise on IR for T2DM patients in this study is scientific and novel, and findings of their combined effect from activating different ways of glucose utilization are expected to benefit both T2DM and other chronic metabolic diseases. Finally, we plan to further investigate protein expression related with glucose uptake in skeletal muscle tissue to elucidate the mechanism of the combined effect on IR in mice in our future study.

China is currently facing a growing threat from NCDs, and it shows no sign of abating. Prevention of NCDs including diabetes through promoting healthy eating and lifestyle has been elevated to a national public policy priority [2, 43]. In October 2016, the State issued the “Healthy China 2030” bringing the NCD issue into sharper and more concrete focus [44]. The findings from this study of vitamin D supplementation on IR for Chinese T2DM patients can also contribute to the alleviation of T2DM epidemic worldwide.

## Trial status

Active protocol version number: 1.3; January 1, 2019. The details of the protocol versions with the date of the amendment are provided in Table 3. Recruitment began on April 1, 2018. Currently, recruitment is ongoing and is expected to be completed in December 2020, and intervention and the last follow-up are expected to be completed in December 2021.

**Table 3:** Protocol versions

Version	Date and changes
1.0	March 7, 2017, original protocol
1.1	March 1, 2018, introduction and background was improved
1.2	July 15, 2018, amendments of the inclusion and exclusion criteria
1.3	January 1, 2019, more comprehensive plan of the analysis was incorporated

## Data Availability

Not applicable.

## Abbreviations

ABI: Ankle-brachial index
ANOVA: Analysis of variance
AMPK: AMP-activated protein kinase
CG: Control group
DXA: Dual-energy X-ray absorptiometry
EG: Endurance exercise group
GLUT-4: Glucose transportor-4
HbA1c: Glycated hemoglobin
HOMA: Homeostatic model assessment
IR: Insulin resistance
MHR: Maximal heart rate
OGTT: Oral glucose tolerance test
T2DM: Type 2 diabetes mellitus
VD: Vitamin D
VEG: Vitamin D and endurance exercise group
25(OH)D: 25-Hydroxyvitamin D
